# Multimodal Wearable Monitoring of Exercise in Isolated, Confined, and Extreme Environments: A Standardized Method

**DOI:** 10.3390/mps9010015

**Published:** 2026-01-21

**Authors:** Jan Hejda, Marek Sokol, Lydie Leová, Petr Volf, Jan Tonner, Wei-Chun Hsu, Yi-Jia Lin, Tommy Sugiarto, Miroslav Rozložník, Patrik Kutílek

**Affiliations:** 1 Faculty of Biomedical Engineering, Czech Technical University in Prague, 27201 Kladno, Czech Republic; marek.sokol@cvut.cz (M.S.); lydie.leova@fbmi.cvut.cz (L.L.); petr.volf@fbmi.cvut.cz (P.V.); tonnejan@fel.cvut.cz (J.T.); patrik.kutilek@cvut.cz (P.K.); 2 Graduate Institute of Biomedical Engineering, National Taiwan University of Science and Technology, Taipei 106, Taiwan; wchsu@mail.ntust.edu.tw; 3 Graduate Institute of A.I. Cross-Disciplinary Technology, National Taiwan University of Science and Technology, Taipei 106, Taiwan; yijia@mail.ntust.edu.tw; 4 Industrial Technology Research Institute, Hsinchu 310, Taiwan; tommysugiarto@itri.org.tw; 5 Hydronaut Project a.s., 15500 Prague, Czech Republic; miroslav.rozloznik@hydronaut.eu

**Keywords:** wearable sensors, electromyography, inertial measurement units, exercise monitoring, methods, confined environments

## Abstract

This study presents a standardized method for multimodal monitoring of exercise execution in isolated, confined, and extreme (ICE) environments, addressing the need for reproducible assessment of neuromuscular and cardiovascular responses under space- and equipment-limited conditions. The method integrates wearable surface electromyography (sEMG), inertial measurement units (IMU), and electrocardiography (ECG) to capture muscle activation, movement, and cardiac dynamics during space-efficient exercise. Ten exercises suitable for confined habitats were implemented during analog missions conducted in the DeepLabH03 facility, with feasibility evaluated in a seven-day campaign involving three adult participants. Signals were synchronized using video-verified repetition boundaries, sEMG was normalized to maximum voluntary contraction, and sEMG amplitude- and frequency-domain features were extracted alongside heart rate variability indices. The protocol enabled stable real-time data acquisition, reliable repetition-level segmentation, and consistent detection of muscle-specific activation patterns across exercises. While amplitude-based sEMG indices showed no uniform main effect of exercise, robust exercise-by-muscle interactions were observed, and sEMG mean frequency demonstrated sensitivity to differences in movement strategy. Cardiac measures showed limited condition-specific modulation, consistent with short exercise bouts and small sample size. As a proof-of-concept feasibility study, the proposed protocol provides a practical and reproducible framework for multimodal physiological monitoring of exercise in ICE analogs and other constrained environments, supporting future studies on exercise quality, training load, and adaptive feedback systems. The protocol is designed to support near-real-time monitoring and forms a technical basis for future exercise-quality feedback in confined habitats.

## 1. Introduction

Isolated, Confined, and Extreme (ICE) environments are settings that combine isolation from the outside world, confinement within limited living and working spaces, and exposure to extreme physical conditions. Examples include spaceflight, Antarctic stations, submarines, and simulated analog space missions. These environments impose unique physical and psychological stressors and therefore provide valuable models for studying human performance and well-being under unusual conditions.

A frequently discussed challenge in ICE environments is maintaining crew physical fitness [1,2]. Many missions involve long-term stays, such as prolonged submarine patrols or overwintering at polar stations, where limited space, equipment constraints, and environmental stressors can lead to health risks, physical deconditioning, and decreased performance, potentially jeopardizing mission success [3]. Regular physical activity plays a critical role not only in preserving musculoskeletal and cardiovascular function, but also in supporting sleep quality, mood, stress regulation, immune function, and biological rhythms [4,5,6,7].

On the International Space Station (ISS), structured exercise represents a cornerstone countermeasure against physiological deconditioning during prolonged exposure to microgravity, including losses of muscle and bone mass, reductions in muscle strength, and decreased aerobic capacity. Crews follow agency-specific exercise protocols using specialized hardware [7,8]. Recent evidence further suggests that higher-intensity, lower-volume exercise prescriptions may achieve comparable physiological benefits while reducing time demands on crew members [9].

The importance of maintaining physical fitness extends beyond spaceflight to other ICE environments, including Antarctic expeditions [2,10], high-altitude operations [11,12], and speleological missions [13]. In submarine settings, exercise options are constrained by limited space, restricted equipment, and atmospheric conditions such as reduced oxygen availability and elevated carbon dioxide levels. Available equipment typically includes cycle ergometers, steppers, treadmills, climbing devices, and simple resistance tools such as weight vests or elastic bands [5]. Extended submarine deployments have been associated with physiological deconditioning [5,14], reduced aerobic capacity [3], and increased body fat [15].

Effective training in such constrained environments benefits from professional guidance, including feedback on exercise technique, appropriate load progression, and strategies to support adherence, as well as from monitoring training load to reduce the risk of overtraining and injury [16,17]. However, systematic assessment of exercise execution and physiological response in ICE environments remains challenging.

Traditionally, muscle function in sports, exercise, and rehabilitation has been evaluated using relatively simple quantitative measures [18,19]. While these approaches are practical, they often depend strongly on participant effort, motivation, and experience, which may compromise measurement reliability [20]. Wearable physiological sensors offer an opportunity to complement such assessments by providing objective, continuous measures of neuromuscular and cardiovascular responses during exercise.

Recent work has summarized physiological sensing approaches applied in ICE contexts, including wrist-worn accelerometers, electrocardiographic patches for heart rate variability assessment, and inertial measurement units for tracking movement [21]. However, these studies typically focus on individual sensing modalities and do not describe an integrated, reproducible method that combines sensor placement, exercise selection, signal synchronization, normalization procedures, and data processing for exercise monitoring in confined environments.

Prior physiological monitoring in ICE contexts has often relied on isolated modalities (e.g., accelerometry/IMU or ECG patches) and has rarely provided an end-to-end, operationally specified framework that is reproducible in confined habitats. This limits cross-mission comparability, prevents standardized within-subject tracking, and makes it difficult to build reliable real-time coaching or quality-assessment tools. Here we address these gaps by providing a standardized, space-efficient method that (1) defines a confined-habitat exercise panel, (2) specifies wearable sensor placement and acquisition settings for simultaneous sEMG–IMU–ECG measurement, (3) implements repetition-level synchronization and segmentation (video-verified boundaries), (4) applies MVC-based normalization enabling within- and between-session comparisons, and (5) provides a reproducible processing and feature-extraction pipeline suitable for near-real-time monitoring. Together, these components form a practical foundation for reproducible exercise-quality assessment and adaptive feedback in ICE analog missions.

In addition, exercise assessment methods intended for ICE missions should be evaluated under conditions that closely reflect real operational constraints, such as confined spaces with limited equipment availability. Analog missions provide a controlled yet realistic framework for testing physiological, psychological, and technical aspects of human performance in simulated ICE environments [13,22]. Such missions may take place in diverse settings, including underwater habitats, deserts, caves, mountainous regions, or polar environments, and are well suited for protocol development and verification [22].

Therefore, this article describes and demonstrates the operational feasibility of a reproducible multimodal method for monitoring exercise execution in ICE analog environments using wearable surface electromyography, inertial, and cardiac sensors. The method specifies exercise selection, sensor configuration, normalization to maximum voluntary contraction, synchronization across data streams, and feature extraction, and its feasibility is demonstrated through application during multiple analog missions conducted in the Hydronaut DeepLabH03 habitat.

## 2. Materials and Methods

The proposed exercise-monitoring method was implemented and evaluated in the DeepLabH03 habitat, a simulated ICE environment. The research project No. TM04000062 was approved by a Hydronaut ethical committee (no. 012023) and this study was performed in line with the principles of the Declaration of Helsinki.

### 2.1. DeepLabH03

**Figure 1:**
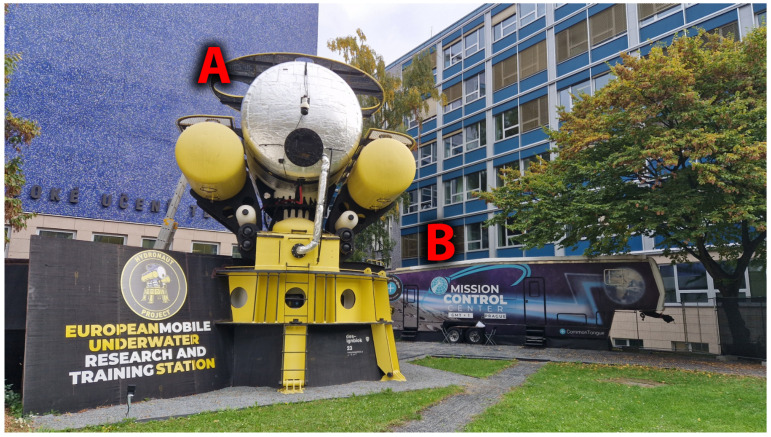
Area of the Little Moon City Prague complex used for the experiment. Exercise sessions were performed inside the DeepLabH03 habitat (A) and were remotely supervised from the Mission Control Center (B).

The DeepLabH03 research and training facility (Figure 1) is designed for short- and long-term stays in ICE environments. This mobile habitat can be deployed underwater or used for surface operations. It supports a wide range of studies—from the effects of ICE conditions on human psychology and physiology to technology testing under extreme constraints.

DeepLabH03 is located on the Czech Technical University (CTU) campus in Prague as part of the Little Moon City Prague (LMC) complex, which also includes life-support systems, a Mission Control Center (MCC), and an EVA area.

The pressurized volume is ∼20 m^3^ with ∼8 m^2^ of usable floor space, accommodating up to three crew members (see [22]). Ventilation is provided by a fresh-air, single-pass system, and the crew is continuously monitored by MCC.

### 2.2. Missions

To test the proposed training programs and verify the functionality of the system for measuring surface electromyography (sEMG) and movement data under ICE analog conditions, a total of sixteen missions had been conducted by September 2025. Prior to each mission, all participants underwent medical screening and training. Written informed consent was obtained from all participants, who were informed of potential risks and could withdraw at any time without penalty.

The goals of these missions were to evaluate the feasibility and operational applicability of the proposed exercise-monitoring method, specifically:

- feasibility and ergonomics of the wearable system for sEMG and motion data acquisition,
- applicability of the exercise protocol under confined and resource-limited conditions,
- operational readiness of the habitat and technical infrastructure for method execution during analog missions.

This article reports findings from Mission 15, which lasted 7 days and was conducted in September 2024. The monitored training procedure was implemented on Days 4 and 5 of the mission, allowing for initial environmental adaptation before physiological measurements began.

### 2.3. Participants

Three healthy right-handed adults (N=3) participated in the study carried out during Mission 15: mean age 40.0±8.7 years, mean height 170.3±6.8 cm, and mean body mass 69.2±22.4 kg. The corresponding body-mass index (BMI) was 23.5±5.6 kg·m^−2^ (P1: 95 kg, 178 cm, 45 y, BMI ≈30.0; P2: 56 kg, 165 cm, 30 y, BMI ≈20.6; P3: 56.5 kg, 168 cm, 45 y, BMI ≈20.0).

All participants were informed about the experimental procedures and provided their written consent.

### 2.4. Exercise Selection and Design

The exercise set was designed in stages. A literature review identified modalities suitable for isolated, confined, and extreme (ICE) environments and summarized monitoring approaches and operational constraints (pressure, temperature, humidity, space, gravity). Because existing methods were not directly transferable, we developed and tested customized routines for the DeepLabH03 habitat.

From a broader catalogue of movements performed by participants (see Table A1), we retained ten exercises for downstream analyses to balance lower-/upper-body and core demands, cover a spectrum of intensities, and remain feasible in ICE conditions with minimal equipment. The selection emphasized (i) physiological diversity—i.e., distinct heart-rate (HR) and surface electromyography (sEMG) profiles enabling statistical contrasts across tasks, (ii) fitness-maintenance relevance in ICE conditions, and (iii) operational suitability (limited space, simple setup).

This panel (Table 1) collectively elicits varied cardiopulmonary and neuromuscular stimuli: dynamic, aerobic-biased drills yield higher HR, slow-tempo resistance tasks produce high sEMG at moderate HR, isolated upper-limb work maintains strength with low HR, while core stability and rotation challenge trunk musculature with distinct activation patterns. These choices align with evidence that combined aerobic+resistance modalities preserve fitness in confinement and that lower-/upper-limb resistance and core training counteract deconditioning in ICE analogs [23,24,25,26,27,28,29].

Together, the selected exercises form a standardized and operationally feasible panel intended for method-based assessment of exercise execution and physiological response in confined environments.

### 2.5. Technology Selection and Design

#### 2.5.1. Monitoring of Physiological Parameters

The Diana mission [22] demonstrated the value of continuous monitoring of physiological parameters, providing a comprehensive data set and valuable insights into crew adaptation to the isolated underwater environment and early detection of signs of health problems. These physiological metrics (ECG, respiratory rate, and electrodermal activity, all sampled at 500 Hz), allow assessment of both short-term responses and long-term trends in crew health and performance. From an exercise perspective, these parameters are key to assessing cardiovascular workload, stress response, and recovery effectiveness. They not only allow monitoring of the body’s immediate responses to physical activity, but also long-term trends in adaptation to training. A detailed description of the multisensor device is provided in [22].

#### 2.5.2. Monitoring of Muscle and Movement Activity

**Figure 2:**
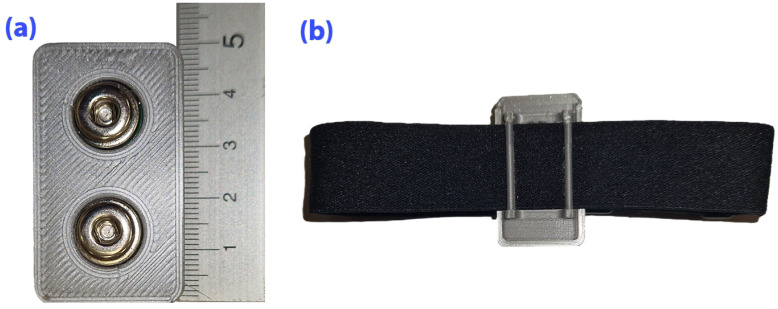
Wearable sEMG/IMU sensor module used in the proposed method. (**a**) Skin-facing side of the device with two snap connectors, to which disposable gel electrodes are attached for sEMG signal acquisition. (**b**) Outer side of the device showing the elastic fixation strap, which enables firm attachment of the sensor to the limb and minimizes the risk of electrode detachment and motion-related artifacts during exercise.

A wearable system was developed to measure muscle potentials over target muscles and motion data in ICE conditions (Figure 2); it records sEMG via an ADS1292 AFE at 1 kHz over SPI (with START, DRDY/INT, RESET) and motion via an LSM6DSO gyroscope/accelerometer and a QMC5883L magnetometer on I^2^C at 100 Hz. The device is built on a 40 × 22 mm PCB around an ESP32-S3-MINI-1U-N8 that handles sensor communication, on-board processing, and 2.4 GHz wireless transmission (up to 20 dBm); two electrode inputs are conditioned by analog filtering and routed to the ADS1292 (the unused input tied to mid-supply), a flexible on-board antenna provides Wi-Fi, and a USB-C connector supports data transfer and battery charging. Power is supplied by a single-cell 3.7 V Li-Po (40 × 20 × 3 mm) regulated to 3.3 V. The system is described in more detail in [21].

The setup was used in a preliminary feasibility phase to verify the multimodal acquisition system under ICE analog conditions. Although the sEMG module used the ADS1292 front end (typically for ECG), it yielded stable recordings sufficient to evaluate muscle activation patterns.

The sensing module was designed as a low-cost, rapidly deployable prototype to assess the feasibility of the monitoring architecture. Its specifications (1 kHz sampling, ADS1292 AFE) were selected to balance power consumption and signal fidelity for biofeedback applications. While absolute voltage calibration was not the objective, preliminary signal inspection confirmed the presence of characteristic sEMG spectral components (20–150 Hz) and responsiveness to load, sufficient for the relative analyses presented in this study.

### 2.6. Testing Method Design

The proposed measurement systems are used to evaluate muscle activity and movement patterns in defined training programs. Sensor placement follows recommendations in [30] and was adapted to ICE constraints to target key muscle groups. Sensors are attached via disposable self-adhesive electrodes and, if needed, secured with medical tape or strapping.

Before exercise, each participant performs Maximum Voluntary Isometric Contractions (MVC) for all studied muscles to normalize sEMG. Each MVC consists of 3 s rest, 3 s maximal contraction, and 3 s rest, executed with a team member providing resistance in the required direction. Recommendations for sEMG electrode placement and the corresponding MVC tasks are listed in Table 2.

### 2.7. Signal Processing

Data (sEMG 1 kHz, IMU 100 Hz, ECG/EDA/Respiration rate 500 Hz) were streamed in real-time to an InfluxDB time-series database. After aligning all streams to exercise start/stop, exercise videos were reviewed and repetition onsets/offsets were manually annotated; these timestamps partitioned signals into repetition-specific segments.

**Figure 3:**
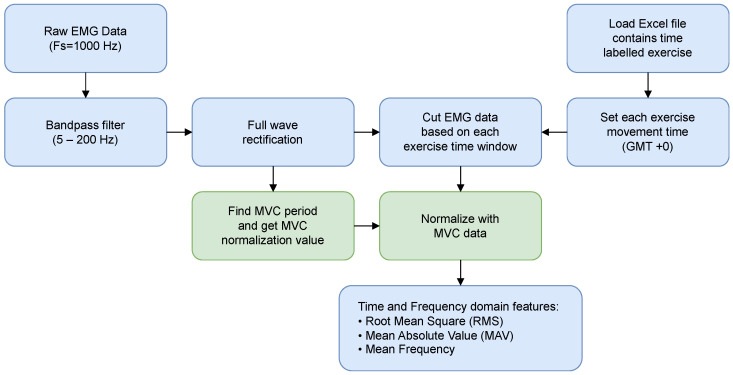
PC-side pipeline for sEMG signal processing.

sEMG was band-pass filtered (5–200 Hz), full-wave rectified, and processed identically for MVC trials. For each muscle, the MVC peak/plateau provided the normalization reference, enabling between-session/participant comparisons [30]. Using the labeled windows, sEMG segments were normalized to MVC, yielding dimensionless activation traces [31]. From these, standard features were computed: root mean square (RMS), mean absolute value (MAV), and mean frequency (Figure 3). Mean frequency quantifies the average frequency content of the sEMG power spectrum over the entire segment and is computed as the weighted mean of the spectral frequency bins.

For HRV, ECG was band-pass filtered (0.5–40 Hz) and segmented by the same timestamps. RR intervals were derived with NeuroKit2 [32]. HR was the segment mean; time-domain HRV used root mean square of successive differences (RMSSD) from artifact-corrected NN intervals.

### 2.8. Statistical Analysis

Statistical analyses were designed to evaluate within-subject effects and method sensitivity, rather than to support population-level inference.

Specifically, the objective was to determine whether the proposed method can reliably differentiate exercises and muscle activation patterns within individuals under repeated measurements, consistent with the fixed-effects focus typical of feasibility studies in ICE mission contexts.

All analyses were conducted in R (version 4.5.1). Linear mixed-effects models (LMMs) were employed to account for the repeated-measures design and the hierarchical structure of the data. Separate models were estimated for electromyographic outcomes and heart rate variability indices.

When applying linear mixed-effects models to small-N, repeated-measures designs, an important methodological consideration is the interpretation of random effects. A common recommendation is that random effects should have at least five levels to allow reliable estimation of variance components [33]. However, recent studies indicate that models with fewer levels can still yield unbiased fixed-effect estimates when random effects are treated as nuisance parameters rather than quantities of inferential interest [33]. In our study, we used an LMM with only three participants as the random-effect levels, acknowledging that while this limits the precision of variance estimates, it does not substantially affect the accuracy or uncertainty of fixed-effect estimates. Thus, following previous simulation-based findings, using fewer than five random-effect levels, as in our case, can be acceptable when the focus is on fixed effects rather than the random-effect structure.

For sEMG outcomes, root mean square and mean absolute value were log-transformed prior to analysis, while mean frequency was analyzed on the original scale. Fixed effects comprised the current exercise performed (‘exercise condition’), the specific muscle group monitored (‘muscle’), and the day of training (‘day’), including relevant interactions. Random intercepts were specified for participants, and in sEMG models, additional random effects were included to account for within-participant variability across muscles. For HRV outcomes, including mean N–N interval and root mean square of successive differences, fixed effects consisted of exercise condition and day, with participants modeled as random intercepts.

To identify the most appropriate model, competing model specifications were compared using indices of model performance, including conditional and marginal $R^{2}$, intraclass correlation coefficient, root mean square error, residual standard deviation (sigma), as well as information-theoretic criteria and their associated weights. An overall performance score was also considered to balance model fit and complexity.

For each fitted model, an omnibus analysis of variance (ANOVA) was conducted to evaluate the significance of fixed effects. The use of ANOVA on the linear mixed-effects models allowed for a global test of the significance of fixed effects before proceeding to pairwise comparisons. This approach reduces the risk of inflating Type I error by first establishing whether an overall effect of exercise condition, muscle, or day was present. Only when these tests indicated significant main effects or interactions were post-hoc comparisons between exercise conditions conducted. From the selected models, estimated marginal means were computed, and pairwise comparisons between exercise conditions were conducted with Tukey adjustment to control for multiple testing. Statistical significance was set at p<0.05. The exercise conditions comprised the selected exercises and their corresponding codes listed in Table 1.

Linear mixed models assumption checks were performed using visual inspection. This included assessment of the normality of residuals and random effects, verification of linearity between predictors and outcomes, inspection of homogeneity of variance across fitted values, and evaluation of potential multicollinearity among predictors. Model diagnostics were further used to confirm that the final models provided a robust representation of the data.

Descriptive summaries and graphical representations were generated to complement the statistical analyses. These included plots of estimated marginal means with confidence intervals and participant-level trajectories to visualize consistency of responses across exercise conditions and individuals.

### 2.9. End-to-End Method Summary

To ensure reproducibility and clarify how the individual methodological components are integrated into a unified system suitable for isolated, confined, and extreme (ICE) environments, the complete end-to-end workflow of the proposed method is summarized below:

1. *Exercise panel selection for confined habitats.* A set of exercises was selected to be feasible in space-limited environments and executable with minimal equipment (Table 1).
2. *Standardized sensor placement and MVC tasks.* Wearable sEMG, IMU, and ECG sensors were placed according to predefined anatomical landmarks, and maximum voluntary contraction (MVC) tasks were performed to enable signal normalization (Table 2).
3. *Multi-modal data acquisition and streaming.* All sensor modalities were recorded simultaneously and streamed in real time to a central database (InfluxDB), ensuring synchronized acquisition across devices with defined sampling rates for each modality.
4. *Temporal synchronization and repetition segmentation.* Exercise repetitions were segmented at the repetition level using video-verified temporal markers, enabling precise alignment of sEMG, IMU, and ECG signals across modalities.
5. *sEMG preprocessing and normalization.* sEMG signals were filtered, rectified, and normalized to MVC, followed by extraction of time-domain features characterizing neuromuscular activation (Figure 3).
6. *ECG preprocessing and HRV computation.* ECG signals were processed to detect R–R intervals, from which heart rate variability (HRV) indices were computed using a standardized analysis pipeline.
7. *Integrated outputs for feasibility assessment.* The resulting synchronized multi-modal features were used to verify method feasibility and data quality, and establish a methodological foundation for future real-time exercise quality assessment and adaptive feedback in ICE environments.

Real-time operation in this study refers to synchronized data acquisition, streaming, and storage with the possibility of on-the-fly visualization, while repetition segmentation was performed offline using video-verified temporal boundaries.

## 3. Method Verification and Feasibility Outcomes

The preliminary analyses provide an initial evaluation of muscle activation patterns and autonomic responses during structured exercise protocols during Mission 15.

### 3.1. Inter-Individual Variability and Consistency

**Figure 4:**
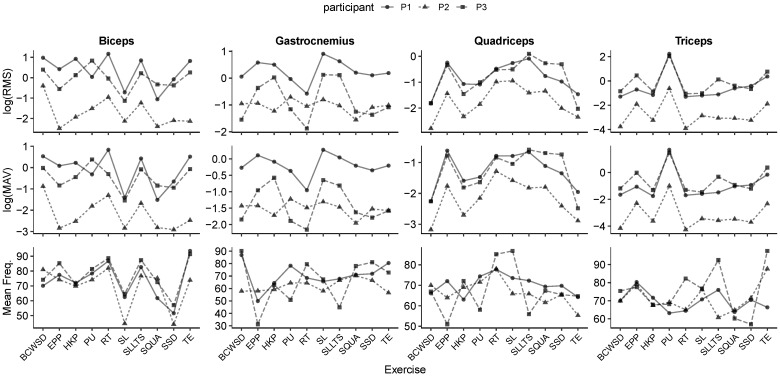
Participant-level trajectories of sEMG parameters (RMS, MAV, and mean frequency) across exercise conditions, stratified by muscle. Lines represent participant-specific means, and points indicate observed values, allowing visualization of inter-individual consistency and within-muscle variability.

Across all exercise conditions, participant-level trajectories of sEMG parameters (see Figure 4) revealed both consistent activation patterns and notable inter-individual variability. RMS and MAV values generally followed similar trends across muscles, with the biceps and triceps exhibiting the highest activation levels, while quadriceps and gastrocnemius responses were more variable. Despite this variability, the directionality of responses across exercise conditions was largely preserved, suggesting that the wearable system captured physiologically meaningful patterns rather than noise.

When comparing across participants, some muscles (particularly the biceps) showed stable activation magnitudes with relatively narrow dispersion, indicating reproducible recruitment strategies. In contrast, gastrocnemius activation displayed wider variability between individuals, reflecting differences in technique or muscle recruitment strategies. Similarly, MAV distributions highlighted that participant P3 tended to produce higher amplitude responses across most exercises, while P1 and P2 showed more moderate but consistent activation.

Frequency-domain measure demonstrated greater consistency across participants compared to amplitude-based indices. This suggests that while the intensity of activation varied between individuals, the underlying spectral characteristics of muscle activation were more stable across participants. Such consistency reinforces the potential of frequency-based indices as robust markers for monitoring adaptation in ICE environments.

### 3.2. Electromyographic Responses Across Exercise Conditions

Linear mixed-effects modelling revealed significant effects of exercise condition on both amplitude- and frequency-domain sEMG indices. Specifically, for RMS and MAV, omnibus tests indicated that the main effect of *exercise* was not significant (RMS: F(9, 223)=1.59, p=0.118; MAV: F(9, 223)=1.68, p=0.096), but there were robust *exercise × muscle* interactions (RMS: F(27, 223)=2.38, p<0.001; MAV: F(27, 223)=2.43, p<0.001), suggesting muscle-specific modulation across exercise modes. In contrast, frequency-domain responses (Mean Freq.) exhibited a strong main effect of exercise (F(9, 223)=3.46, p<0.001) as well as a significant *exercise × muscle* interaction (F(27, 224)=4.15, p<0.001). Day of testing was also a significant covariate in the amplitude-domain models (RMS: p=0.014; MAV: p=0.012), but not for mean frequency (see Table 3 for full omnibus test results).

**Figure 5:**
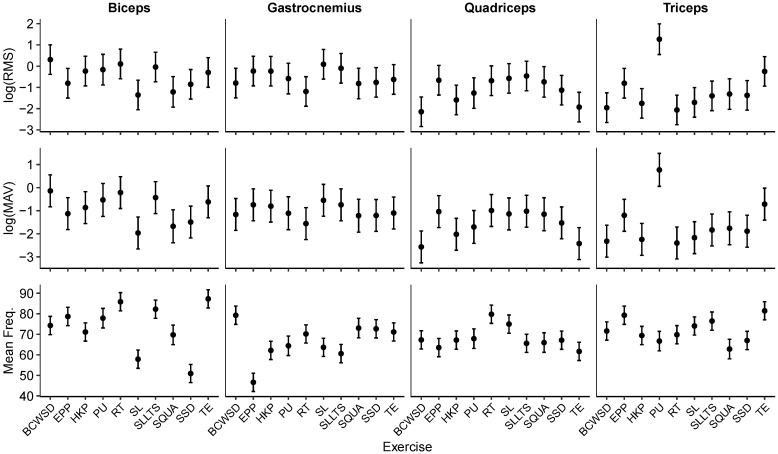
Estimated marginal means (EMMs) of sEMG parameters (RMS, MAV, and mean frequency) across exercise conditions. Each panel displays group-level means with standard errors, separated by muscle where applicable. Pairwise comparisons between conditions were adjusted using Tukey’s method.

Estimated marginal means further illustrate these patterns (Figure 5). RMS and MAV responses were consistently lower during squat (SQUA) and single lunge (SL) compared to baseline conditions, particularly in the quadriceps and triceps, whereas push-up (PU) and elbow plank + pointe (EPP) elicited significantly higher triceps responses. For frequency-domain measures, mean frequency showed pronounced reductions during single lunge (SL) and slower speed deadlift (SSD), but elevations for exercises emphasizing upper-limb loading, such as triceps extension (TE). These muscle- and task-specific divergences reinforce the necessity of interpreting amplitude- and frequency-domain indices in tandem.

To provide transparency while maintaining concision, only a subset of the results is presented here. Full pairwise contrasts for each muscle and condition are available in Table A2. Together, these detailed post-hoc comparisons show that while some exercise contrasts did not reach significance, others exhibited robust differences that align with expected biomechanical demands.

### 3.3. Heart Rate Variability Responses

Linear mixed-effects modeling of HRV indices revealed limited exercise-related effects, though day of testing emerged as an important covariate for selected parameters. For MeanNN, the omnibus test indicated no main effect of exercise condition (F(7, 44.9)=1.75, p=0.123), but a significant effect of day (F(1, 46.1)=8.32, p=0.006), with lower values observed on subsequent test days. The effect of day reflects a temporal sequence within the mission rather than a specific day-of-week or external environmental condition, as measurements were conducted on consecutive mission days under comparable habitat settings following initial adaptation.

**Figure 6:**
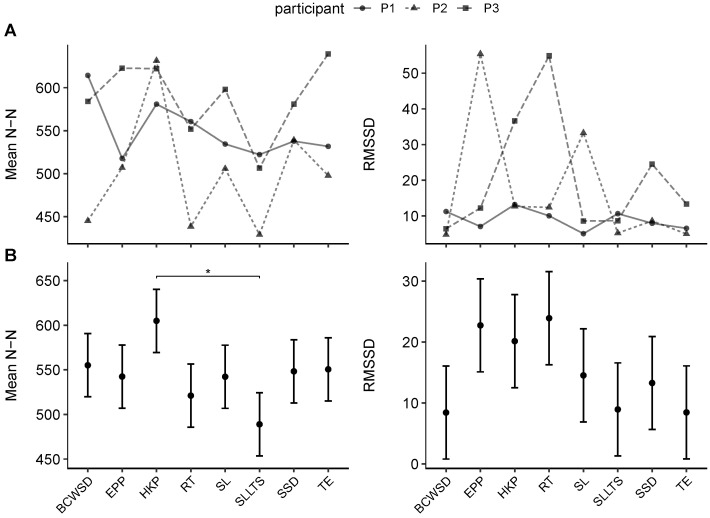
(**A**) Participant-level trajectories of HRV indices (MeanNN and RMSSD) across exercise conditions. Lines represent individual participants, with markers indicating mean values per condition, highlighting both within- and between-subject variability in cardiac autonomic responses. (**B**) Estimated marginal means of HRV indices (MeanNN and RMSSD) across exercise conditions. Group-level means with standard errors are shown, illustrating condition-specific differences in autonomic modulation (* p<0.05).

Post hoc pairwise comparisons identified a significant difference between the high knee pulls (HKP) and single lunge L + triple shoulder press (SLLTS) conditions (p=0.035), with higher MeanNN during HKP. All other contrasts were non-significant after Tukey adjustment. Estimated marginal means are shown in Figure 6, panel B, and full omnibus results are summarized in Table 4.

RMSSD values demonstrated no significant differences between exercise conditions (F(7, 45.1)=0.79, p=0.599) or days (F(1, 45.5)=0.20, p=0.658). Although point estimates suggested elevated RMSSD during elbow plank + pointe (EPP) and Russian twist (RT), variability was high, and no pairwise contrasts reached significance after Tukey adjustment.

HRV indices are known to be highly sensitive to segment length and stationarity. Because our exercise bouts were short and dynamic, and segments were defined at the repetition/exercise level, large and consistent RMSSD changes across conditions were not expected. The isolated MeanNN contrast (HKP vs. SLLTS) may reflect differences in immediate cardiovascular load during compound versus dynamic movements rather than a stable autonomic signature.

### 3.4. Feasibility and Ergonomic Evaluation of Wearable System

Field measurements showed that an integrated sensing approach is feasible in ICE environments. The wearable sEMG–IMU system captured stable muscle-activation patterns and body kinematics during dynamic and resistance exercises, while ECG added complementary insight into cardiovascular load and recovery.

Based on experience from earlier missions, the configuration was reduced from seven to four sensors on the dominant limb, which improved comfort and workflow without compromising the detection of task-related changes in muscle activity. MVC-based normalization supported between-session and between-subject comparisons despite minor placement variability. sEMG and IMU signal quality was sufficient to monitor execution and patterns consistent with fatigue, and the joint use of sEMG amplitude features with IMU angular-velocity profiles enabled interpretation of both effort and movement strategy.

## 4. Discussion

This study demonstrates the feasibility of a wearable approach for monitoring exercise execution in an ICE analog habitat and provides preliminary evidence that such a system can capture physiologically meaningful patterns under realistic operational constraints. The results align with emerging research advocating multimodal wearable sensors for real-time training feedback and activity monitoring [34,35,36].

To clarify how the proposed method advances beyond prior studies conducted in isolated, confined, and extreme (ICE) environments, we explicitly map commonly reported methodological limitations in the literature to the corresponding contributions of this work:

- *Single-modality physiological monitoring.* Previous ICE studies typically focus on isolated sensing modalities (e.g., IMU-based activity tracking or heart rate monitoring), which limits the interpretation of exercise quality and physiological load. In contrast, the proposed method integrates synchronized sEMG, IMU, and ECG measurements with shared temporal segmentation, enabling concurrent assessment of neuromuscular, kinematic, and cardiovascular responses.
- *Non-standardized sensor placement and missing normalization.* Earlier approaches often rely on ad hoc sensor placement and raw or minimally processed signals, reducing reproducibility across sessions. Here, sensor placement is explicitly defined and combined with MVC-based normalization, enabling within- and between-subject comparisons under confined-habitat constraints (Table 2).
- *Lack of reproducible synchronization and processing pipelines.* Many prior studies do not fully specify signal synchronization, preprocessing, and feature extraction steps, hindering replication. In this work, a fully described, multi-modal processing pipeline is provided, including filtering, normalization, and feature extraction for each sensing modality.
- *Limited suitability for confined habitats.* Exercise procedures and hardware setups commonly used in laboratory environments are often impractical in space- and equipment-limited ICE settings. The proposed method was explicitly designed for confined spaces and minimal equipment and was validated in an ICE analog habitat.

Together, these elements position the proposed method as a reproducible, end-to-end framework that addresses key methodological gaps in existing ICE exercise monitoring studies and establishes a foundation for future real-time exercise quality assessment.

### 4.1. Methodological Considerations and Internal Verification

Consistent with the project’s feasibility focus, the sEMG module was not benchmarked against a laboratory-grade reference at this stage. Instead, internal verification confirmed stable recordings, physiologically plausible envelopes, and congruent activation onsets relative to task demands [21]. While this approach is sufficient for preliminary interpretation of activation timing and relative changes, a formal validation and characterization of noise floor, linearity, and frequency response remain priorities for the refinement phase.

### 4.2. Physiological Responses

A preliminary finding of this study is the system’s ability to differentiate exercises based on their distinct muscle activation profiles. While a uniform main effect of exercise on sEMG amplitude (RMS, MAV) was not observed, the analysis revealed robust and highly significant *exercise × muscle* interactions (p<0.001 for both RMS and MAV), consistent with prior evidence that exercise effects on sEMG are muscle-specific [37]. This result is not a limitation but rather a confirmation of the system’s validity; it correctly identified that the impact of an exercise is dependent on the specific muscle being monitored. For instance, as detailed in the supplementary data, triceps activation during push-ups was significantly greater than during seven other exercises (p<0.001), quantitatively confirming the biomechanical demands of this upper-body task. This specificity is crucial for ensuring that training programs in ICE environments effectively target the intended muscle groups to counteract deconditioning. Notably, this aligns with recommendations emphasizing that wearable systems should detect meaningful differences between exercise modalities and support actionable interpretation [38]. The observed contrasts also reflect the purposeful inclusion of varied movement types: dynamic (high knee pulls), compound (lunge plus shoulder press), and isolated (biceps curls), which are known to elicit distinct sEMG responses, as demonstrated in wearable-sensor research by Majumder et al. (2017) [29].

Furthermore, the significant main effect of exercise on mean frequency suggests the system is sensitive to more than just activation intensity. Mean frequency reflects the spectral characteristics of the sEMG signal, which are influenced by motor unit firing rates and recruitment strategies. The observed differences between exercises, such as the pronounced shifts during slow-tempo movements like the Slower Speed Deadlift, indicate that the system can detect changes in neuromuscular strategy. This capability could be leveraged in future applications to monitor fatigue [39], assess movement quality, or guide exercise tempo in real-time.

In contrast to the clear sEMG results, heart rate variability indices showed limited differentiation between exercise conditions, likely due to short bout duration and small sample size. However, the lower heart rate on the second testing day suggests possible habituation to the procedure. While condition-specific HRV effects remain preliminary, ECG integration provides a foundation for future studies.

In addition, the limited and inconsistent modulation of HRV indices observed across exercise conditions is consistent with known physiological constraints of HRV analysis. Time-domain HRV metrics, particularly RMSSD, are typically evaluated over longer, quasi-stationary segments, such as resting baseline or post-exercise recovery periods, whereas short, dynamic exercise bouts are characterized by increased signal variability and reduced sensitivity to exercise-specific autonomic modulation.

Accordingly, the integration of ECG in the present study primarily serves to demonstrate synchronized acquisition and analysis feasibility within the multi-modal method rather than to derive definitive exercise-specific HRV signatures. Future iterations of the method will therefore incorporate standardized pre-exercise baseline recordings (2–5 min), defined post-exercise recovery windows (e.g., 1–3 min), and, where feasible, controlled breathing conditions to improve the interpretability and robustness of HRV-based outcomes in confined habitats.

### 4.3. Feasibility, Ergonomics, and Practical Implications in ICE Environments

Operationally, the system functioned reliably within the DeepLabH03 habitat, with real-time synchronization and on-the-fly processing achievable during exercise blocks, in line with prior studies that reported comparable real-time performance of wearable sEMG–IMU systems in constrained settings [36,40]. Importantly, reducing the configuration from seven to four sensors on the dominant limb improved comfort and workflow without compromising the detection of task-related changes, in agreement with [41], an essential trade-off in confined missions where setup time and user burden must be minimized. These observations reinforce the practicality of lean sensor sets for routine crew training, provided that placement and normalization to %MVC are consistent.

### 4.4. Limitations and Future Directions

Key limitations include the small sample size (N=3) and the absence of simultaneous validation against a commercial reference system. While the sample size is characteristic of specialized ICE analog missions, it restricts the generalization of the specific physiological findings to a broader population. Importantly, while this sample size limits population-level generalization, it is well aligned with the operational realities of ICE missions, where crew sizes are inherently small and data collection prioritizes individual monitoring and longitudinal assessment over cross-sectional inference.

The high inter-individual variability observed in sEMG amplitudes (Figure 4) highlights that while the system reliably captures data, the biological response is highly individual. Consequently, the statistical results should be interpreted as a demonstration of the method’s sensitivity to detect changes within subjects, rather than as normative values for these exercises. The exercise set, while representative for feasibility, was intentionally compact. Future studies will expand participant numbers and mission days to strengthen inference, extend bout durations and recovery controls to resolve HRV dynamics, and perform device-level validation against a certified sEMG system. In addition, we plan to develop a feedback module that functions as a virtual coach to motivate participants and improve movement quality. This module will classify exercises and assess execution quality by comparing sensor-derived features against a curated reference dataset, delivering real-time, actionable feedback and adapting targets to individual baselines and mission constraints.

## 5. Conclusions

In an ICE analog habitat, a compact, wearable system captured muscle-specific activation patterns and provided multi-modal context for exercise evaluation under realistic operational constraints. Despite limited sample size, amplitude–frequency sEMG analyses revealed interpretable, task-dependent signatures, and the streamlined four-sensor configuration improved comfort without evident loss of informational value. While formal device validation is pending, internal verification and consistent physiological responses support the system’s feasibility for monitoring execution quality and training load in confined missions. Next steps will establish quantitative hardware validation, scale participation across missions, and develop real-time feedback mechanisms to enable reliable, autonomous guidance for maintaining crew fitness in isolated, confined, and extreme environments.

## Figures and Tables

**Table 1:** Exercise abbreviations, descriptions, and physiological impact (HR/sEMG) with literature support.

Abbr.	Exercise	Brief Description	Physiological Impact & Support
HKP	High Knee Pulls	Fast, in-place knee lifts engaging hip flexors and core.	Aerobic stimulus (elevates HR), moderate sEMG in hip flexors/quadriceps; supports cardiovascular maintenance in confinement [23,28].
SQUA	Slowest Speed Squats	Slow-tempo bodyweight squats; emphasis on control/time-under-tension.	High sEMG quadriceps/glutes, moderate HR; mitigates lower-limb deconditioning in bed-rest/spaceflight analogs [24,25].
SSD	Slower Speed Deadlift	Hip-hinge deadlift with band/bodyweight targeting posterior chain.	High sEMG hamstrings/glutes/lumbar extensors, moderate HR; posterior-chain strength in ICE analogs [27].
PU	Push-Ups (slow)	Controlled push-ups for chest/shoulders/triceps.	High sEMG pectorals/triceps, moderate HR; upper-body strength maintenance in confinement [26].
SL	Single Lunges	Alternating lunges challenging lower-limb strength and stability.	High sEMG quadriceps/glutes, moderate HR; complements squats; effective against atrophy [24,25].
BCWSD	Biceps Curls (slow eccentric)	Elastic-band curls emphasizing slow lowering phase.	High sEMG biceps, low–moderate HR; band-based resistance suitable for confined spaces [27].
TE	Triceps Extensions	Elastic-band extensions targeting triceps.	High sEMG triceps, low HR; supports upper-limb function maintenance [26].
EPP	Elbow Plank + Pointe	Forearm plank with alternating toe points for added core demand.	High sEMG core (rectus abdominis/obliques), low–moderate HR; psycho-physiological benefits in isolation [28].
RT	Russian Twist	Seated trunk rotations (with/without load).	High sEMG obliques, moderate HR; rotational core stimulus distinct from static planks [28].
SLLTS	Single Lunge L + Triple Shoulder Press	Left lunge combined with three overhead band presses.	High sEMG quadriceps/glutes/deltoids; higher HR due to compound nature; full-body stimulus in limited space [23,27].

**Table 2:** Table of sEMG placement recommendation and corresponding MVC tasks used for muscle-specific normalization.

No.	Muscle	MVC Task	Procedure
1	Gastrocnemius	Calf raise vs. resistance	Perform a calf raise at maximal effort while another person applies resistance in the opposite direction to prevent foot movement.
2	Rectus Femoris	Knee extension vs. hold	Sit with feet off the ground and extend the lower leg forward with maximal effort; another person holds the shank to prevent movement.
3	Triceps Brachii	Elbow extension vs. resistance	Perform elbow extension at maximal effort while another person resists in the opposite direction to prevent arm movement.
4	Biceps Brachii	Elbow flexion vs. hold	Perform elbow flexion at maximal effort while another person holds the arm to prevent movement.

**Table 3:** Omnibus ANOVA results from linear mixed-effects models of sEMG indices.

Index	Effect	df	F	*p*	Significance
RMS	Exercise	9, 223	1.59	0.118	n.s.
	Muscle	3, 6	1.35	0.346	n.s.
	Day	1, 224	6.11	0.014	*
	Exercise × Muscle	27, 223	2.38	<0.001	***
MAV	Exercise	9, 223	1.68	0.096	n.s.
	Muscle	3, 6	1.16	0.402	n.s.
	Day	1, 224	6.49	0.012	*
	Exercise × Muscle	27, 223	2.43	<0.001	***
Mean Freq.	Exercise	9, 223	3.46	<0.001	***
	Muscle	3, 6	3.83	0.073	^†^
	Day	1, 218	0.39	0.534	n.s.
	Exercise × Muscle	27, 224	4.15	<0.001	***

*Notes.* df = degrees of freedom (Kenward–Roger), reported as numerator, denominator; F = F-statistic from the Type III omnibus test of the mixed model; *p* = two-sided *p*-value. Significance: n.s. p≥0.10; ^†^ p<0.10; * p<0.05; *** p<0.001.

**Table 4:** Omnibus ANOVA results from linear mixed-effects models of HRV indices.

Index	Effect	df	F	*p*	Significance
MeanNN	Exercise	7, 44.98	1.75	0.123	n.s.
	Day	1, 46.07	8.32	0.006	**
RMSSD	Exercise	7, 45.11	0.79	0.599	n.s.
	Day	1, 45.55	0.20	0.659	n.s.

*Notes.* df = degrees of freedom (Kenward–Roger), reported as numerator, denominator; F = F-statistic from the Type III omnibus test of the mixed model; *p* = two-sided *p*-value. Significance: n.s. p≥0.10; ** p<0.01.

## Data Availability

The datasets generated and analyzed during the current study are not publicly available due to restrictions imposed by the Institutional Ethics Committee for participant protection and the risk of re-identification given the small number of participants, but are available from the corresponding author on reasonable request.

## Abbreviations

The following abbreviations are used in this manuscript:

ANOVA	Analysis of variance
BCWSD	Biceps curls (slow eccentric)
BMI	Body mass index
CTU	Czech Technical University
ECG	Electrocardiography
EDA	Electrodermal activity
EMMs	Estimated marginal means
EPP	Elbow plank + pointe
EVA	Extravehicular activity
HKP	High knee pulls
HR	Heart rate
HRV	Heart rate variability
ICE	Isolated, confined, and extreme
IMU	Inertial measurement unit
LMC	Little Moon City Prague
LMM	Linear mixed-effects model
MAV	Mean absolute value
MCC	Mission Control Center
MeanNN	Mean N–N interval
MVC	Maximum voluntary isometric contraction
PU	Push-ups (slow)
RMS	Root mean square
RMSSD	Root mean square of successive differences
RT	Russian twist
sEMG	Surface electromyography
SL	Single lunges
SLLTS	Single lunge L + Triple shoulder press
SSD	Slower speed deadlift
SQUA	Slowest speed squats
TE	Triceps extensions

## Appendix A

**Table A1:** Complete list of conducted exercises.

Section	Exercise Name	Reps/Times
A. Warm Up	Ballistic stretch right	1 set 8 reps/set
	Ballistic stretch left	1 set 8 reps/set
	High knee pulls	1 set 4 reps/set
	Hip ER	1 set 4 reps/set
	Hip IR	1 set 4 reps/set
	ITB left	1 set 20 s/set
	ITB right	1 set 20 s/set
	Neck rotation	1 set 20 s/set
	Shoulder backward rotation	1 set 8 reps/set
	Wrist and ankle rotation	1 set 15 s/set
B. Squat	Single bottom half squats	1 set 8 reps/set
	Single squats	1 set 8 reps/set
	Slow down quick up squat	1 set 8 reps/set
	Quick down slow up squat	1 set 8 reps/set
	Continue bottom half squats	1 set 16 reps/set
	Wide single bottom half squats	1 set 8 reps/set
	Wide single squat	1 set 8 reps/set
	Wide slow down quick up squat	1 set 8 reps/set
	Wide quick down slow up squat	1 set 8 reps/set
	Wide continue bottom half squats	1 set 16 reps/set
	Stretch quadriceps muscle	1 set 20 s/set
	Stretch hamstring muscle	1 set 20 s/set
C. Back	Slower speed deadlift	1 set 8 reps/set
	Slower speed row	1 set 8 reps/set
	Slower speed high pull	1 set 8 reps/set
	Single row	1 set 8 reps/set
	Triple row	1 set 4 reps/set
	Single row + Stand on toes	1 set 8 reps/set
	Single row (backhand)	1 set 8 reps/set
	Triple row (backhand)	1 set 4 reps/set
	Stretch upper back	1 set 20 s/set
D. Pectoral and Shoulder	Single push ups	1 set 8 reps/set
	Hands walking push ups	1 set 6 reps/set
	Side raise	1 set 8 reps/set
	Rotator	1 set 8 reps/set
	Pec Deck	1 set 4 reps/set
	Stretch Pectorals muscles	1 set 20 s/set
E. Lunges	Single lunges	1 set 8 reps/set
	Slow down quick up	1 set 8 reps/set
	Quick down slow up	1 set 8 reps/set
	Single bottom half lunges	1 set 8 reps/set
	Stretch quadriceps muscle	1 set 20 s/set
	Stretch hamstring muscle	1 set 20 s/set
F. Triceps and Biceps	Biceps curls	1 set 8 reps/set
	Biceps curls with slower down	1 set 8 reps/set
	Alternating single biceps curls	1 set 8 reps/set
	Pulse single biceps curls	1 set 8 reps/set
	Triceps extensions	1 set 8 reps/set
	Pulse & Triceps extensions	1 set 16 reps/set
	Elbow extension R/L	1 set 8 reps/set
	Triceps hands on ground	1 set 8 reps/set
	Stretch triceps	1 set 20 s/set
	Stretch biceps	1 set 20 s/set
G. Core	Abdominal crunch	1 set 8 reps/set
	Figure 4 crunch left	1 set 8 reps/set
	Figure 4 crunch right	1 set 8 reps/set
	Elbow hand plank	1 set 30 s/set
	Elbow plank	1 set 10 reps/set
	Side plank L/R	1 set 30 s/set
	Sit-up with dumbbell	1 set 8 reps/set
	Russian twist	1 set 8 reps/set
	Walkout plank	1 set 8 reps/set
	Deadbug	1 set 8 reps/set
	Stretch abdominal muscles	1 set 20 s/set
H. Lunges and Shoulder	Single lunge L + sh.press	1 set 8 reps/set
	Single lunge R + sh.press	1 set 8 reps/set
	Single lunge L + triple sh.press	1 set 4 reps/set
	Single lunge R + triple sh.press	1 set 4 reps/set
	Lunges + Rotator L/R	1 set 4 reps/set
	Stretch leg muscle	1 set 20 s/set
	Stretch shoulder	1 set 20 s/set
I. Cool Down	Adductor stretch left/right	1 set 20 s/set
	Hamstrings & Calf left/right	1 set 20 s/set
	Shoulder stretch (thumb down) left/right	1 set 20 s/set
	Triceps left/right	1 set 20 s/set
	Wrist extension left/right	1 set 20 s/set
	Wrist flexion left/right	1 set 20 s/set
	Glute stretch left/right	1 set 20 s/set
	Back stretch	1 set 20 s/set
	ITB2 left/right	1 set 20 s/set
	Quadriceps stretch	1 set 20 s/set
	Abdominal stretch	1 set 20 s/set

**Table A2:** Significant Tukey-adjusted pairwise estimated marginal means (EMMs) contrasts from linear mixed-effects models of three sEMG parameters. EMMs were computed for exercises within each muscle, and pairwise differences were Tukey-adjusted; degrees of freedom follow the Kenward–Roger method. Estimates for RMS and MAV are on the log scale; Mean Freq. is on the original scale. (* p<0.05; ** p<0.01; *** p<0.001; **** p<0.0001.)

Parameter	Muscle	Contrast	Estimate	SE	df	*t*	*p*-Value	Sig.
RMS	Triceps	BCWSD –PU	−3.22	0.66	223.12	−4.910	0.0001	***
	Triceps	HKP–PU	−3.02	0.66	223.12	−4.598	0.0003	***
	Triceps	PU–RT	3.33	0.66	223.12	5.072	<0.0001	****
	Triceps	PU–SL	2.97	0.66	223.12	4.531	0.0004	***
	Triceps	PU–SLLTS	2.67	0.66	223.12	4.062	0.0027	**
	Triceps	PU–SQUA	2.58	0.68	223.00	3.793	0.0072	**
	Triceps	PU–SSD	2.64	0.66	223.12	4.025	0.0031	**
MAV	Triceps	BCWSD–PU	−3.09	0.64	223.11	−4.811	0.0001	***
	Triceps	HKP–PU	−3.01	0.64	223.11	−4.690	0.0002	***
	Triceps	PU–RT	3.17	0.64	223.11	4.932	0.0001	***
	Triceps	PU–SL	2.94	0.64	223.11	4.573	0.0003	***
	Triceps	PU–SLLTS	2.60	0.64	223.11	4.051	0.0028	**
	Triceps	PU–SQUA	2.53	0.67	223.00	3.801	0.0070	**
	Triceps	PU–SSD	2.66	0.64	223.11	4.135	0.0020	**
Mean Freq.	Biceps	BCWSD–SSD	23.38	5.94	223.03	3.937	0.0043	**
	Biceps	EPP–SL	20.79	5.94	223.03	3.500	0.0196	*
	Biceps	EPP–SSD	27.76	5.94	223.03	4.675	0.0002	***
	Biceps	PU–SL	19.97	6.19	223.43	3.227	0.0457	*
	Biceps	PU–SSD	26.94	6.19	223.43	4.354	0.0008	***
	Biceps	RT–SL	27.95	5.94	223.03	4.707	0.0002	***
	Biceps	RT–SSD	34.93	5.94	223.03	5.882	<0.0001	****
	Biceps	SL–SLLTS	−24.35	5.94	223.03	−4.100	0.0023	**
	Biceps	SL–TE	−29.38	5.94	223.03	−4.948	0.0001	***
	Biceps	SLLTS–SSD	31.32	5.94	223.03	5.274	<0.0001	****
	Biceps	SSD–TE	−36.35	5.94	223.03	−6.122	<0.0001	****
	Gastrocnemius	BCWSD–EPP	32.65	5.94	223.03	5.499	<0.0001	****
	Gastrocnemius	EPP–RT	−23.57	5.94	223.03	−3.968	0.0038	**
	Gastrocnemius	EPP–SQUA	−26.42	6.19	223.43	−4.269	0.0012	**
	Gastrocnemius	EPP–SSD	−26.05	5.94	223.03	−4.386	0.0007	***
	Gastrocnemius	EPP–TE	−24.54	5.94	223.03	−4.132	0.0020	**
